# Neonatal renal venous and arterial thrombosis

**DOI:** 10.1186/1824-7288-41-S1-A24

**Published:** 2015-09-24

**Authors:** Mario Motta

**Affiliations:** 1 Neonatology and Neonatal Intensive Care Unit, Children's Hospital of Brescia, Italy

## 

Renal vein thrombosis (RVT) is one of the more common forms of neonatal thrombosis accounting for 15-20% of systemic thromboembolism[1,2]. A median age at presentation of 2-3 days have been reported [1,3], with unilateral and bilateral involvement of renal vein in 89-72% and 28-11% of cases, respectively[4,5]. The clinical presentation of RVT consists of increased kidney size, macro- or microhematuria, reduced urine output with some degree of renal failure and thrombocytopenia. RVT in neonates is a multifactorial disease and it was associated with coagulopathy, maternal diabetes, birth asphyxia, sepsis, placement of central venous lines (CVL) and hereditary thrombophilic factors[5,6]. In specific, FV 1691G>A mutation and elevated Lp(a) concentrations are independent risk factors for the development of RVT in neonates. In non-CVL-related RVT, thrombus formation occurs in the small vessels of the kidney and may extend into the renal vein and inferior vena cava. In the neonates with CVL-related RVT, the thrombus most likely formed first in the vessel adjacent to the CVL and then extended into the kidney[4]. Renal ultrasonography shows uni- or bilateral kidney enlargement with loss of cortico-medullary differentiation and reduced or absent flow in renal vein[7]. Long-term functional complications include renal insufficiency, renal tubular dysfunction and hypertension in up to 30% of patients[1,8]. Controlled data on appropriate clinical management of neonatal RVT are lacking and recommendations of its treatment are based on low quality evidence. Current guidelines suggest for unilateral RVT in the absence of renal impairment or extension into the inferior vena cava, either 1) supportive care with radiologic monitoring for extension of thrombosis or 2) anticoagulation with unfractionated heparin (UFH)/low-molecolar-weight heparin (LMWH) in therapeutic doses rather than no therapy[9]. For unilateral RVT that extends into the inferior vena cava, anticoagulation with UFH/LMWH for a total duration of between 6 weeks and 3 months is suggested[9]. For bilateral RVT with evidence of renal impairment, anticoagulation with UFH/LMWH or initial thrombolytic therapy with tissue plasminogen activator followed by anticoagulation with UFH/LMWH is suggested[9].

Renal artery thrombosis (RAT) in the neonate is far less common than RVT, and there is little information about its incidence. It is associated with umbilical catheters, patent ductus arteriosus and hereditary thrombophilia [10,11]. The clinical presentation is rather silent and renal ultrasound findings can be minimal unless Doppler imaging is used. The outcome of the affected kidneys is poor with common global atrophy. For neonates with a peripheral arterial catheter-related thrombosis, immediate removal of the catheter and UFH anticoagulation with or without thrombolysis or surgical thrombectomy and microvascular repair with subsequent heparin therapy are suggested[9].
